# Overcoming the Limitations of Protein A: Evolution of Bacterial Protein-Based and Synthetic Affinity Ligands for High-Performance IgG Purification

**DOI:** 10.3390/ijms27177618

**Published:** 2026-08-25

**Authors:** Larisa N. Ikryannikova, Mikhail N. Tereshin, Milena V. Baskova, Kristina P. Telepenina, Neonila V. Gorokhovets, Daniel R. Bayzigitov, Eugenia A. Gurylina, Vasiliy N. Stepanenko, Tatiana D. Melikhova

**Affiliations:** 1 I.M. Sechenov First Moscow State Medical University, Institute of Translational Medicine and Biotechnology, 8/2 Trubetskaya Str., 119991 Moscow, Russia; tereshin_m_n@staff.sechenov.ru (M.N.T.); milena.baskova@yandex.ru (M.V.B.); telepenina_kristina@mail.ru (K.P.T.); gorokhovets_n_v@staff.sechenov.ru (N.V.G.); stepanenko_v_n@staff.sechenov.ru (V.N.S.); tdm-63@yandex.ru (T.D.M.); 2 M.V. Lomonosov Institute of Fine Chemical Technologies, MIREA—Russian Technological University, Vernadskogo Pr. 86, 119571 Moscow, Russia; 3 R-Pharm Group Pharmaceutical Company, 119421 Moscow, Russia; bayzigitov@rpharm.ru (D.R.B.); ea.gurylina@rpharm.ru (E.A.G.); 4 M.M. Shemyakin-Ovchinnikov Institute of Bioorganic Chemistry, Russian Academy of Sciences, Miklukho-Maklaya Str. 16/10, 117437 Moscow, Russia

**Keywords:** affinity chromatography, mAbs purification, IgGs, immunoglobulin-binding proteins (IBPs), protein A, hybrid proteins

## Abstract

Monoclonal antibodies (mAbs) are widely used as therapeutic molecules for the treatment of serious diseases, primarily cancer. The market for mAbs is one of the fastest-growing segments of the biopharmaceuticals industry. Purification is a crucial stage in the production of mAbs. While staphylococcal protein A (SpA) affinity chromatography remains the gold standard in industrial mAb purification, its limitations—low alkaline stability and insufficient binding capacity of SpA, as well as the need for harsh acidic elution conditions—have driven extensive efforts for novel progressive affinity ligands. This review focuses on the development and performance of bacterial protein-based affinity resins for the purification of class G immunoglobulins (IgGs), including conventional proteins A and G, the promising protein L, and the more recently discovered protein M (from *M. genitalium*), each offering unique specificities for different antibody fragments and species. Hybrid ligands combining domains from multiple bacterial proteins are also discussed, along with next-generation synthetic alternatives such as affibodies, affimers, nanobodies, etc., as well as peptide-based or mixed-mode ligands. The key finding is that the reliable and time-tested resins like those based on protein A continue to dominate the market, although future trends also point toward smaller, more stable, and cost-effective synthetic ligands for specific applications.

## 1. Introduction

Monoclonal antibodies (mAbs) are widely used as therapeutic molecules for the treatment of significant diseases, primarily cancer. Due to their effectiveness, the market for mAbs is showing impressive growth worldwide and is one of the fastest-growing segments of biopharmaceuticals [1]. The global market for mAbs in 2024–2025 is estimated at more than $250 billion, and within ten years it is projected to reach over $600 billion. As a result of the high competition in this industry, there is an increasing demand for new ways to produce high-quality and cost-effective mAbs. One of the most crucial stages in the production of mAbs is purification of the antibodies. Affinity chromatography on sorbents with immobilized staphylococcal protein A is the most expensive and time-consuming step in this process [1,2,3,4].

The use of the *Staphylococcus aureus* protein A (SpA) in affinity chromatography of immunoglobulins has a long history. Since 1978, when the first commercialized prototype of SpA-based resin was proclaimed, the specifications for these adsorbents used in industrial applications, such as alkaline tolerance, high binding capacity, and low ligand leakage, have steadily improved [5,6,7,8]. For over forty years, the quality of modern protein A-derived affinity resins has improved by several orders of magnitude compared to those based on the native staphylococcal protein. For example, the dynamic binding capacity (DBC_10_) of one of the highest performing resins today, Toyopearl^®^ AF-rProtein A HC-650F, is between 65 and 70 mg/mL at a residence time (RT) of 5 min. In the case of MabSelect PrismA^TM^ X, which is often considered the best sample in the market of affinity resins, this value exceeds 80 mg/mL at RT 6 min. For comparison, dynamic capacities of the first-generation resins based on wild-type SpA did not exceed 10 to 20 mg/mL in the 1980s and 1990s [7,9,10,11].

There are many tools available to enhance the performance of SpA-based resins. Roughly, these can be divided into two categories: first, those related to the nature of the solid support (matrix) and the method of attaching the SpA-derived ligand onto it, and, second, those related to modifying the ligand itself. The latter involves introducing single mutations into the amino acid sequence of protein A, which changes its structure and properties, as well as using individual protein domains that have the best affinity for immunoglobulin G (IgG) instead of the entire SpA sequence, or increasing the number of binding sites for immunoglobulins by oligomerizing selected domains and elongating the ligand [12,13,14]. The density of the ligand on the surface of the carrier is also controlled to minimize steric hindrance during IgG binding [15].

**Figure 1:**
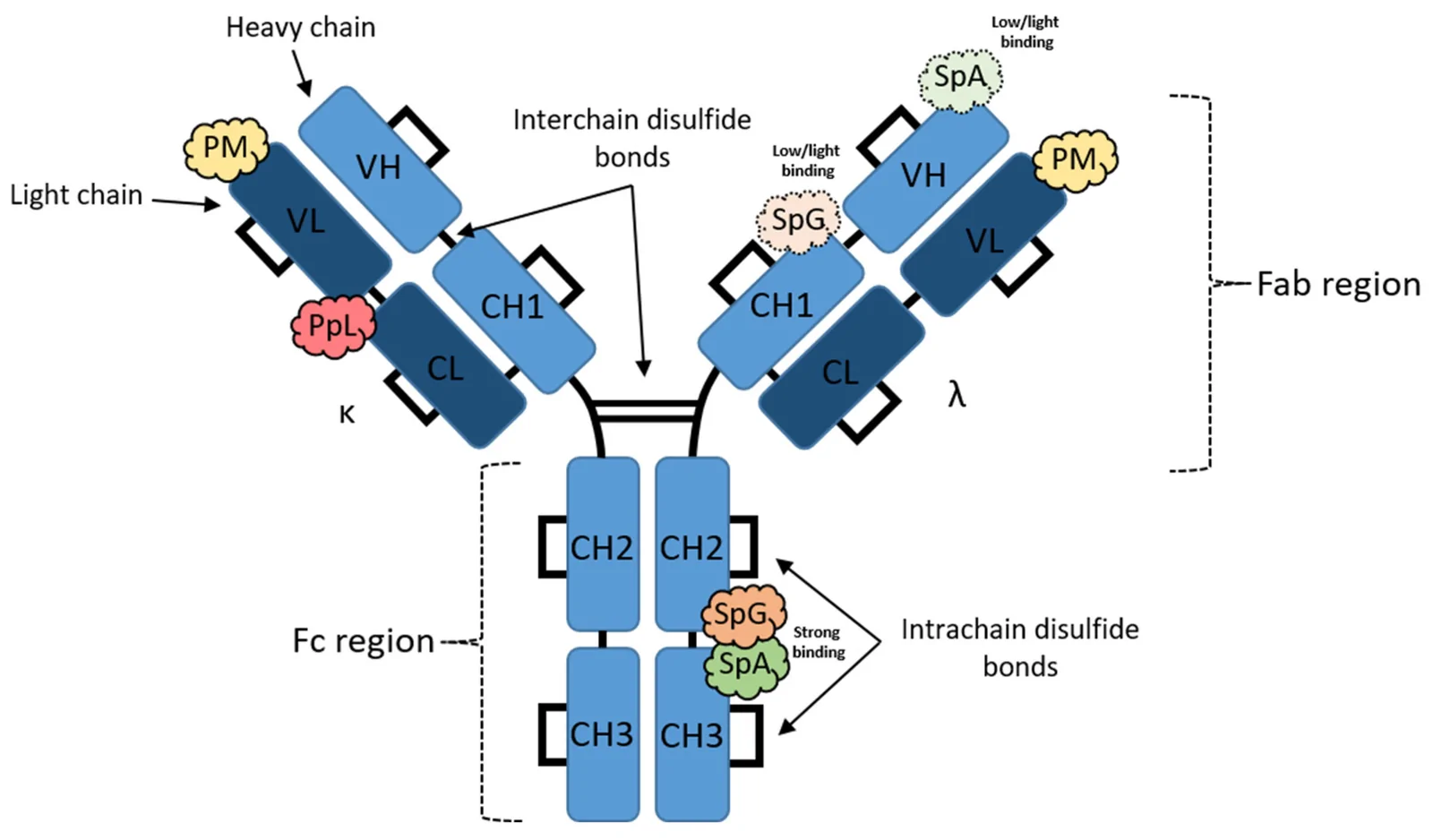
The antibody structure and main binding sites of IgGs affinity ligands.

Other bacterial immunoglobulin-binding proteins (IBPs) have also been developed for use in the affinity chromatography, like protein G (SpG) from *Streptococcus* spp. Note that the protein A or G affinity chromatography is considered as a “gold standard” for purification of mAbs; however, both these proteins bind specifically mostly to the antibody Fc domain, while other antibody fragments such as Fab (fragment antigen-binding) or F(ab′)_2,_ or a single–chain variable fragment (scFv) lacking the Fc domain, cannot be captured effectively enough (Figure 1) [16,17,18,19]. Therefore, proteins with different types of Ig binding sites like protein L and protein M were implemented (Table 1). Protein L from *Peptostreptococcus magnus* (PpL) demonstrates a binding affinity towards the kappa-type light chain of an antibody [20]. The recently discovered protein M has a clear advantage compared to other antibody-binding proteins as it binds to the Fv region of a light chain and therefore is capable of binding to both kappa and lambda light chain types. As a result, it has a very broad scope of binding, including human-, murine-, rat-, rabbit-, goat-, and bovine-derived IgG and human-derived immunoglobulin A (IgA), or immunoglobulin M (IgM), as well as their derivatives such as Fab, F(ab′)_2_ or scFv [21].

The purpose of this review is to discuss the current state of mAb affinity chromatography, specifically focusing on the use of sorbents derived from natural bacterial proteins and their derivatives. We will also consider commercially available variants of these affinity sorbents that are currently presented on the global market. Additionally, possible future directions and prospects for the market development of affinity resins, including those that are based on non-bacterial synthetic ligands, will be discussed.

## 2. Protein A: A Forty-Year Journey in Search of the Perfect

**Figure 2:**
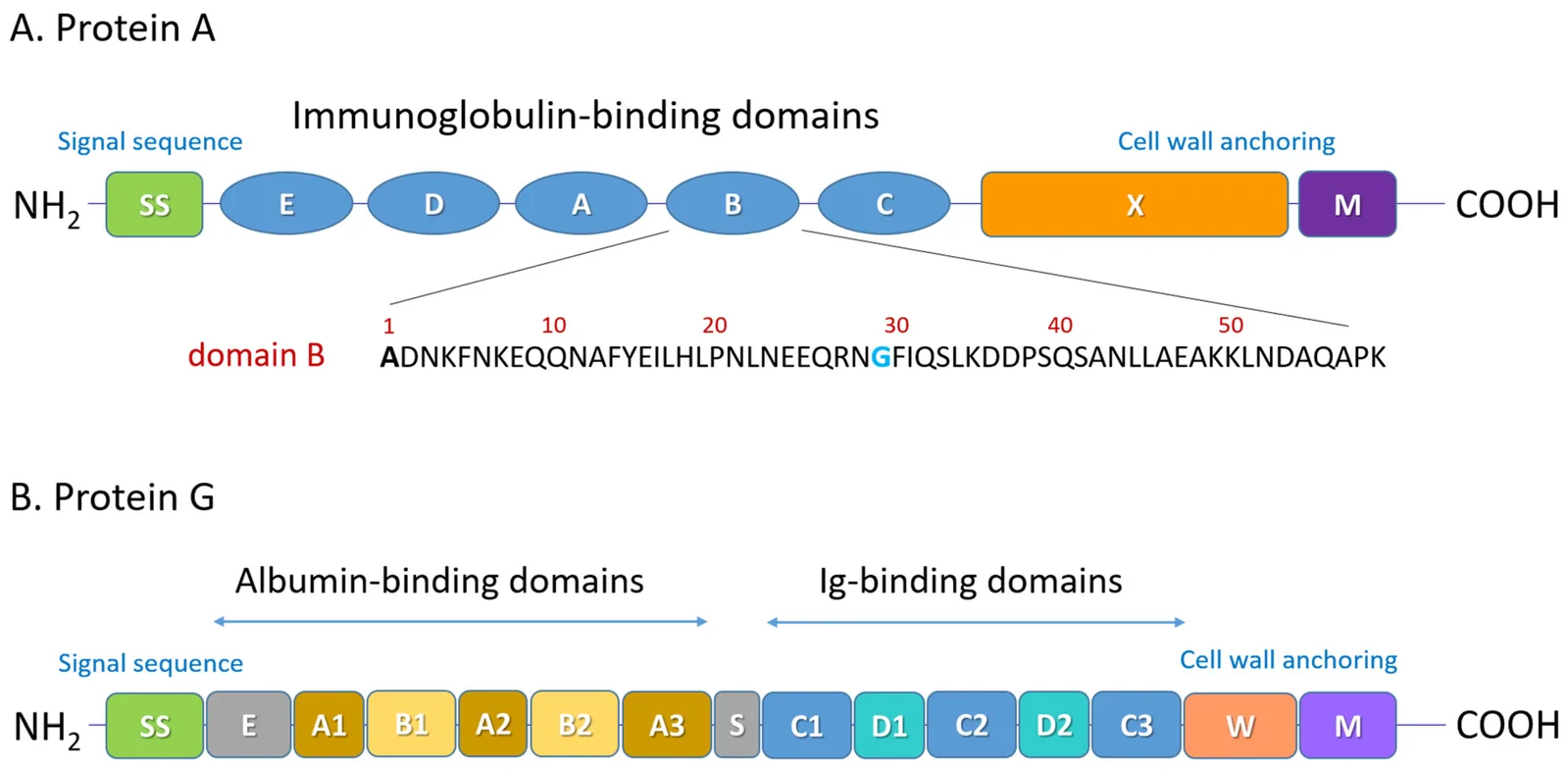
Protein A and protein G schematic structures. (**A**) Staphylococcal protein A: SS—N-terminal signal sequence for the secretion through the membrane; E, D, A, B and C—five homologous IgG-binding domains; X and M regions provide the cell wall attachment. The amino acid sequence of domain B is also shown: the mutation Gly29Ala, which is the key difference between domains B and Z, is highlighted in blue. (**B**) Streptococcal protein G consists of an albumin-binding region and a C-terminal immunoglobulin-binding region. SS—signal sequence; S—spacer separating albumin-binding and immunoglobulin-binding regions. C-terminal W and M sequences anchor the protein to the cell wall.

Protein A is a component on the *S. aureus* surface. It belongs to immunoglobulin binding proteins (IBP) and is believed to protect bacteria against the action of complement system and to decrease the opsonization and phagocytosis that, as a result, allows the microorganisms to evade the influence of the protective factors of the host’s body [36]. SpA is composed of five tandem highly homologous antibody-binding domains (E, D, A, B and C), each consisting of 50–60 amino acids (Figure 2). It also has a 150-amino acid C-terminal region that anchors it to the cell surface. Each domain is made up of three alpha helices arranged in parallel to form a “bundle” structure. Helices 1 and 2 are involved in the binding of SpA to the CH2 and CH3 connection regions of the Fc portion of the antibody, outside of the antigen-binding site. Binding to the Fab part of human IgG on the VH3 region of the variable heavy chain is also possible [10]. The binding sites for the Fab portion are located in Helices 2 and 3, and in between them. Due to the different locations of the sites, one domain can simultaneously bind to the Fc and the Fab parts. All the SpA domains specifically interact with the majority of subclasses of human immunoglobulin G, except IgG3 [7,9,11,23].

The first commercial SpA-based affinity resin was launched in 1978. In the early stages of affinity chromatography, the native protein A isolated from *S. aureus* culture was used. However, this method proved to be commercially unviable, as it had several drawbacks, including high production costs and safety concerns related to working with cultures of pathogenic bacteria. In addition, natural SpA, like other wild-type IBPs, often lacks stability under extreme conditions such as high and low pH [7]. Meanwhile, the operating conditions in industrial antibody purification processes can be quite harsh. For example, eluting antibodies from columns often requires very low pH values (typically pH 3–4) to prevent aggregation or loss of function, while cleaning chromatographic columns and regenerating sorbents between cycles often involves using high concentrations of sodium hydroxide (up to 0.5 M). The binding capacity of the native protein A and SpA-based ligands to antibodies was also far from ideal at that time. For more than forty years, SpA has undergone significant modifications to improve its properties. There are numerous engineered variants of SpA-based ligands that demonstrate enhanced alkaline and chemical stability, as well as a higher binding capacity several times greater than the original, natural protein A [5,6,12]. Technically, protein A modifications have covered several main areas including the use of individual SpA domains instead of the natural five ones, the multimerization of domains, and the insertion of amino acid substitutions into the protein sequence. Different types of matrices are being explored to enhance the mass transfer process and optimize the interaction with antibodies, in order to minimize hindrances caused by steric factors [6,14,37,38,39,40].

The main achievements in improving the properties of the SpA-based ligands are briefly listed in Table 2. The improvement of alkaline stability in SpA-based ligands has been a central focus for a long time, because industrial cleaning-in-place (CIP) protocols routinely use harsh alkaline solutions such as 0.5 M sodium hydroxide to remove contaminants and denatured proteins. Native SpA is inherently unstable under these conditions due to the deamidation of asparagine residues, particularly those located within asparagine–glycine dipeptides, which are highly susceptible to hydrolytic cleavage and conformational degradation. The most labile of these sites in wild-type domains is the Asn–Gly pair at positions 28–29. A major breakthrough occurred in 1987 with the introduction of the Z domain, an engineered variant of the native B domain carrying the single-point mutation G29A, where glycine at position 29 is replaced by alanine. This mutation eliminates the critical Asn–Gly cleavage site and so significantly enhances alkaline tolerance while retaining IgG-binding affinity [18,41]. Other mutations and their combinations have also been investigated (Table 2). Beyond single-point mutations, domain multimerization has proven beneficial: tetrameric and hexameric ligands distribute the impact of alkaline damage across multiple domains, so that even if some binding sites are lost, sufficient capacity remains for effective purification [13,15,38,40,42,43,44].

A major challenge in SpA affinity chromatography is the need for low-pH elution (typically pH 3.0–3.3) to dissociate antibodies from the ligand. Such deeply acidic conditions can induce antibody aggregation, deamidation, conformational changes, and loss of biological activity. In this situation, the introduction of the Z domain carrying the G29A mutation compared to the native B domain also played a positive role. Although primarily designed for alkaline stability, the Z domain also eliminates binding to the VH3 region of the Fab domain, which shifts the elution pH upward compared to wild-type SpA [18]. Then, additional point mutations to further raise the elution pH were also introduced. For instance, the H18S mutation in the Z domain, alone or in combination with N28A, was shown to increase the average elution pH by more than 0.5 units while retaining comparable dynamic binding capacity and selectivity [59]. Another approach involved substituting surface-exposed residues such as Gln10, Asn11, Glu15 or Leu17 with histidine residues. Histidines become protonated in a pH-sensitive manner, and their introduction effectively increases the dissociation rate of the antibody under acidic conditions [46]. A different strategy was the creation of calcium-dependent SpA variants. By inserting a calcium-binding loop between Helix 2 and Helix 3 of the Z domain, researchers generated the Zca variant, which binds IgG only in the presence of calcium ions. Elution is then achieved by simply adding a chelating agent such as EDTA under neutral pH conditions, completely avoiding acidic buffers [47]. Similarly, the so-called Thermal Responsive Protein A (TRPA) was developed, which retains its native structure at 2–10 °C and binds IgG efficiently at low temperatures, but undergoes unfolding at 40 °C, releasing the bound antibody [52]. Overall, all these modifications share a common goal to protect the integrity of the purified antibody by avoiding exposure to pH values below 4.0.

The improvement of binding capacity in SpA affinity chromatography has been a major driver of all innovations, as the native protein A ligand typically utilizes only a fraction of its potential binding sites due to steric hindrance and limited accessibility. Even though native SpA contains five IgG-binding domains, only two to three antibodies can bind simultaneously to one ligand molecule in solution, and the stoichiometry becomes even lower when the ligand is immobilized on porous resin beads [15,38]. The most fundamental approach to increasing the number of functional binding sites per unit volume of resin is the polymerization or multimerization of individual binding domains. By creating homo-multimers of a single domain, such as the B domain, C domain, or the engineered Z domain, the number of available binding sites per ligand molecule is increased. Systematic studies showed that increasing the degree of polymerization improves adsorption capacity up to the octamer level, but beyond that no further gains are observed. Consequently, most popular commercial resins today employ tetrameric to hexameric ligands, typically comprising four to six copies of the B or C domain variants [15]. However, multimerization alone is not sufficient; the density at which these ligands are coupled to the resin matrix is equally critical. When ligand density exceeds approximately twenty mg per milliliter of resin, the availability of binding sites decreases sharply due to overcrowding [38]. Another powerful strategy to overcome the physical limitations of the matrix surface is dextran grafting, in which SpA ligands are attached to dextran chains that are themselves grafted onto agarose gels. The dextran layer provides an extended, flexible three-dimensional adsorption space that allows antibodies to penetrate and bind to ligands in three dimensions rather than only on a two-dimensional surface [53]. Linker engineering between individual domains has also emerged as a promising but still underexplored area. The natural interdomain sequences in SpA are short and highly conserved. Prolonging the linker region with either flexible or rigid sequences could improve domain accessibility and thereby increase binding capacity [15,57,58]. Beyond the ligand itself, the method of immobilization onto the solid support dramatically affects binding capacity. The traditional random immobilization via amino groups using EDC/NHS chemistry results in a random orientation of ligands, with many binding sites facing the matrix and becoming inaccessible. In contrast, site-directed or oriented immobilization could position the ligand in a uniform end-on orientation where all binding sites face the mobile phase [38,54,55,56].

Thus, the most successful modern SpA adsorbents should combine multiple strategies, including rational point mutations to eliminate labile asparagine–glycine sequences, multimerization of domains to enhance binding capacity, proper coupling to solid matrices to create a three-dimensional binding space, optimization of ligand density to avoid steric crowding, oriented immobilization to maximize site accessibility, and provision of relevant elution conditions at weakly acidic to neutral pH to create a robust, alkali-tolerant affinity platform with high binding capacity. In conclusion, it is worth noting that protein A chromatography has become the standard in antibody production, with most commercialized SpA adsorbents being available. Despite this, the search for more advanced versions of this protein is still ongoing. In this regard, an interesting question arises regarding the theoretical limits of protein A capacity. There is reason to believe that the performance of SpA resins can continue to improve. One possibility for modification could be, for example, the elimination of all amino acids that are not required for the structure, expression, or binding of the protein. This would reduce the size of the protein, making it more compact and allowing for an increase in the density of ligands on the beads, without reducing the space available for antibody binding [7]. The number of domains in a SpA molecule, as well as the spacing between domains and the linkers between the matrix and domains, can be varied to optimize the capacity of the resin. There was also an expectation that the creation of hybrid proteins, combining both parts of protein A and other bacterial IBPs, could lead to promising results. This will be discussed in Section 6 concerning hybrid ligands.

## 3. Protein G: A Powerful Competitor or Eternal Benchwarmer?

Protein G is an Ig-binding protein, which was originally isolated from group C and G streptococci [60]. It has the ability to bind to all four subclasses of human and animal IgGs. In this protein, the C-terminal region anchors it to the bacterial cell wall and is also responsible for binding immunoglobulins, while the N-terminal region binds serum albumin and some other proteins (see Figure 2). Depending on the bacterial strain, there are two to three IgG-binding domains, resulting in a molecular weight ranging from 58 to 65 kDa. Each IgG-binding domain consists of approximately 55 amino acid residues folded into a beta-sheet structure, with two antiparallel and two parallel beta strands, with an alpha helix diagonally crossing the sheet.

When compared to protein A, SpG has some advantages, such as broader IgG subclass and species specificity. Another key difference is the nature of its interaction with the Fc region of antibodies. SpA binds to Fc primarily through hydrophobic interactions, while SpG uses mainly polar interactions, including multiple hydrogen bonds and salt bridges. Despite the different types of interaction and the lack of any sequence or structural homology, both proteins compete for the same binding site on the Fc region, specifically the hinge region that links the second and third constant domains of the heavy chain. In addition to Fc binding, SpG is also capable of binding to the Fab region of antibodies, specifically to the constant CH1 domain of IgG1, IgG3, and IgG4, but it does not bind to the Fab of IgG2. However, its binding strength to Fab fragments is not strong enough for efficient and robust purification of human Fabs.

Native SpG has several major limitations. The most significant one is that, in addition to binding to immunoglobulins, it also binds to other naturally occurring proteins such as serum albumin and others, which greatly reduces the efficiency of antibody purification when starting from complex biological sources like cell culture supernatants or blood plasma. Other limitations include low stability under harsh cleaning-in-place conditions and generally lower binding capacities compared to modern engineered commercial SpA variants. As a result, there are only a relatively small number of commercial samples of SpG-based resins being used for antibody isolation and purification on a commercial scale. Nevertheless, SpG affinity chromatography remains the preferred choice for certain specialized applications, such as purifying human IgG3 from serum.

Recombinant versions of SpG that lack these drawbacks are currently under development. One of the first improvements involved deleting the domains responsible for binding non-IgG proteins, primarily albumin, to eliminate unwanted cross-reactivity [61] (Table 3). Another challenge was to enhance the alkaline stability of SpG. To achieve this, the most susceptible residues, such as asparagine and glutamine, were replaced by use of a single-point mutation strategy in the C2 immunoglobulin-binding domain. The most stable version was a double mutant, C2_N736A_, where asparagines at positions 7 and 36 were replaced with alanine [62]. Three specific mutations (Ala24Glu, Lys28Arg, and Val29His) in SpG allowed for the formation of additional hydrogen bonds and salt bridges at the interface of interaction with an antibody, resulting in a five-fold higher binding affinity for rabbit and human immunoglobulins [63]. Later, the same researchers found that introducing additional mutation Lys28His and replacing asparagine at position 35 with a negatively charged amino acid, such as glutamate or aspartate, within the interface of the Protein G/human IgG Fc domain led to a 50-fold change in binding affinity when the pH was changed from mildly acidic to mildly basic. At that, the wild-type interface showed only a negligible change [64]. Further modifications led to the creation of an engineered variant of SpG with eight-point mutations, which significantly improved its affinity for the Fab portion of IgG. Moreover, this interaction can be effectively regulated through pH changes, as there is a ~1000-fold difference in KD when the pH changes from 7.5 to 4 [65]. The opposite task, namely, the elimination of the unwanted ability of the G protein to bind to the Fab region of antibodies, was addressed by the researchers [25]. In their work, a mutant was created, in which Asn478 at the interface with the Fab was replaced with Arg. This resulted in the effective loss of this secondary binding activity while maintaining high affinity for the Ig Fc region.

In summary, SpG has valuable IgG-binding properties, but it is hampered by cross-reactivity, low alkaline stability, and moderate affinity. Recombinant variants of SpG have been successfully generated with improved characteristics through domain deletion, targeted point mutations, and structure-guided design. These engineered versions have higher affinity, enhanced stability, and tunable pH-dependent binding, expanding the usefulness of SpG for antibody purification and allowing it to compete with commercial SpA-based resins.

## 4. Protein L: Exclusivity Is Worth the Price

Protein L is a surface immunoglobulin-binding protein originated from *P. magnus*. It was identified in 1985 by Myhre and Erntell as a membrane protein with broad Ig-binding activity [66], and was found to be capable of binding to the VL regions of κ1, κ3, and κ4 light chains with strong affinity (Kd ≈ 10^−9^ M), but not κ2 and λ subtypes [29]. This unusual binding specificity allows PpL to function as a universal Ig ligand, binding antibodies of all Ig classes as well as single-chain variable fragments (scFv) and Fab fragments [28]. Structurally, PpL consists of four or five, depending on the bacterial strain, highly homologous domains, with a molecular weight of approximately 76–106 kDa. Each domain is composed of a β-sheet formed by four β-strands and a central α-helix. It has been found that PpL binds to antibodies through two distinct binding sites with different affinities. The first site has an affinity that is 25–50 times higher than the second one. The first binding site is formed by 12 amino acid residues, mainly from the second β-strand and α-helix, while the other one involves 14 amino acid residues from the third β-strand and α-helix [29,67].

Despite its unique ability to bind the kappa light chain variable region without interfering with the antigen-binding site, protein L is less commonly used for capture purposes in downstream processing of Fc-containing full-size antibodies compared to protein A. This is primarily due to the fact that commercial protein L-conjugated resins typically have lower binding capacities and require more harsh conditions (i.e., a lower pH value) for elution [68].

Thus, protein L is a unique ligand among other affinity ligands due to its specific interaction with immunoglobulin light chains. However, the current higher market price of PpL compared to SpA or SpG has limited its widespread use. Nevertheless, efforts are being made to develop methods for the efficient and cost-effective production and purification of recombinant PpL [20].

## 5. Protein M: Bet on the Dark Horse

In addition to proteins A (from *S. aureus*) and G (from *Streptococcus* sp.) that bind to the Fc domain of IgGs, and protein L (from *P. magnus*) that targets certain subtypes of the Fv domain of the kappa light chain, one more immunoglobulin-binding protein with remarkable binding properties—protein M from *Mycoplasma genitalium*—was discovered more recently. The cell-wall-less bacteria of the genus *Mycoplasma* are commensal, opportunistic or pathogenic bacteria that can colonize a variety of hosts, including plants, animals or humans. They are classified as mollicutes, a group characterized by their reduced genomes and limited metabolic capabilities. Despite the peculiar metabolism, mycoplasmas are able to efficiently infect hosts and cause a wide range of symptoms, such as fever or inflammation, and diseases like atypical pneumonia, leading to chronic infection. Mycoplasma protein M, the 50 kDa immunoglobulin-binding protein, was first reported in Grover’s study [21]. This protein binds with high affinity to the conserved regions of the Fv light chains, and has a very broad scope, being capable of binding to kappa and lambda light chains of human IgG, IgA, and IgM molecules as well as murine, rat, rabbit, goat, and bovine IgGs. The structure of protein M is different compared to well-known bacterial IBPs like protein A or protein G [21].

By binding to the conserved regions of the Fv domain, protein M sterically blocks the antigen-binding site through its C-terminal domain, which likely protrudes over the antigen-binding site, and inhibits antibody binding to its cognate antigen. The dissociation of the complex between IgG and the wild-type M protein is very slow, and in fact, the formation of this complex is essentially irreversible. This prevents its use as an antibody affinity reagent, unless very harsh elution conditions are used. However, it has been shown that the introduction of eight histidine residues on the Fv binding interface of protein M can promote the dissociation between the protein M and the antibody at low pH and, thus, provide the potential for purifying a wide range of antibody formats (IgG, F(ab′)_2_, Fab, scFv), as well as antibody conjugates [30].

Despite the fact that the antibody purification technology based on protein M is currently the least developed compared to other antibody-binding proteins, the unique binding properties of this protein with Fab fragments open up new possibilities for the development of highly effective antibody purification systems in the biotechnology and pharmaceutical industries. It has been shown that protein M can be a superior affinity reagent for egg yolk immunoglobulin (IgY), and possibly for purifying variable region-based antibodies and fragments (e.g., scFv, Fab or nanobody), as well as unconventional antibodies from reptiles and amphibians. Using protein M affinity chromatography columns, an ~125-fold increase in effective IgY was achieved in the eluate compared to traditional IgY extraction methods [69]. Immobilized on Ni^2+^-modified superparamagnetic nanoparticles, the protein M-8His mutant showed high stability and reusability, retaining 76.1% of its initial activity after storage at 4 °C for 15 days and 90.5% after four consecutive cycles of use. This makes a starting point for introducing the protein M into commercially available antibody purification tools [70].

## 6. Hybrid Ligands: Everything at Once, or Versatility at the Cost of Quality?

In the early stages of the development of effective affinity sorbents, researchers spent some time experimenting with the creation of chimeric protein ligands containing fragments (typically binding sites of immunoglobulins) from various bacterial IBPs. One of the first hybrid proteins designed was the fusion 63 kDa protein AG, which contains all five IgG-binding sites from SpA coupled with three Fc binding domains from SpG. Another variant from the same researchers, the 45 kDa protein ZZC, includes two synthetic domains Z based on the region B of SpA coupled to three native protein G domains. Comparative immunochemical studies have demonstrated that both chimeric products have a broader spectrum towards immunoglobulins of different species and subclasses than the parental protein A and protein G molecules. Furthermore, it has been suggested that these fusion proteins are less pH-dependent compared to SpA or SpG alone [32]. A hybrid protein AG tagged with colloidal gold particles has been developed as a highly versatile and convenient probe for immunochemical and immunocytochemical studies, with broader spectrum towards immunogbobubins of different species and subclasses than the individual parent Fc-binding proteins [71]. A fusion AG protein designed in [35] consisted of a full-length SpA and SpG with a flexible, variable-length peptide linker connecting the Ig-binding domains D from SpA and C1 from SpG respectively. The chimeric proteins obtained bound human and mouse IgG molecules with up to 58-fold higher affinity and broader specificity than the parental binding domains; at that, a longer linker provided the better binding characteristics (Table 4).

In the studies by Kihlberg et al. [72,73], four Ig-binding parts of protein L (B-repeats B1–B4) and two parts of protein G (C1 and C2) were combined to create a hybrid molecule, protein LG. This molecule was found to bind to the large majority of intact human Igs, as well as Fc and Fab fragments and Ig light chains. The binding was specific, and the hybrid molecule proved to be an effective and versatile tool for detection and purifying antibodies and antibody fragments. Similarly, in pursuit of the ideal, unrestricted Ig-binding protein, the authors of [34] have created the hybrid protein LA by fusing four Ig κ light-chain binding domains from PpL with four IgG Fc and Fab-binding regions from SpA. This fusion protein, LA, bound to human Ig from different classes and IgG from a variety of mammalian species. Single-chain Fv (scFv) antibodies containing the κ light chain variable domain or the V_H_III (variable domain of the heavy chain of Ig) determinant were also efficiently purified using immobilized protein LA (Table 4).

It should be noted that the peak of popularity for hybrid proteins occurred during the last decade of the previous century and then began to decline. This can be explained by the increased instrumental capabilities in biotechnology, which have allowed for the development of simpler affinity ligands, such as, for example, synthetic peptide ligands. In addition, most developments over the past 20 years have still focused on optimizing familiar and well-studied SpA-based sorbents. However, some patented variants of sorbents based on hybrid bacterial proteins, including more complex variants such as A, G, and L protein fragment-based ligands (Table 4), are now available on the commercial market (see Table 5).

## 7. Alternative Affinity Ligands for Immunoglobulin Purification

Although protein A-based resins dominate the market, the development of new technologies continues to move away from traditional, natural bacterial IBPs towards a new generation of synthetic and bioengineered alternatives. Recent years have seen the emergence of interesting solutions, for example, alternative scaffold proteins, which are synthetic small and stable protein frameworks. An example of this class of proteins is the affibody molecule. Affibody proteins are 58 amino acid 3-helix bundles based on the Z (B-derived) domain of staphylococcal SpA. Affibody molecules for a range of different targets have been selected from combinatorial libraries through a number of different display methods. The advantage of affibodies is that they are easy to engineer and resistant to mutations. Due to their small size, they can be conveniently produced using solid phase peptide synthesis. Different variants of affibodies have been developed for a variety of applications, including affinity chromatography, particularly for binding specific antibody classes such as IgG, IgA, and IgE. However, these specific proteins are not typically used in the industrial production of immunoglobulins, as their use is often limited to more specialized tasks [76]. Other types of binding scaffolding proteins such as affitins, affimers, nanobodies, monobodies, repebodies or mixed-mode ligands (MML) have also been proposed (Table 6). Affitins directed against the Fc portion of antibodies were engineered based on a small (7 kDa) DNA-binding protein, Sac7d, from the thermoacidophilic archaebacterium *Sulfolobus acidocaldarius*, or related proteins, such as Sso7d from *S. solfataricus*. These ligands are notable for their remarkable tolerance to extreme pH and temperature, making them ideal for use in industrial chromatography, including the purification of human IgG1 antibodies [77]; for diverse protein targets, different affitins can be generated using focused random mutagenesis and in vitro selection via yeast or ribosome display [78]. The newly developed promising alternative to antibodies engineered from the human stefin A protein or from a consensus plant phytocystatin protein sequence is affimer proteins. These proteins are engineered to generate a small, monomeric and highly soluble scaffold molecule with high thermal stability that lacks disulphide bonds and glycosylation sites. Two regions with variable sequence, typically nine residues in length, with randomized amino acid sequences in each, are inserted into the scaffold to provide the binding interface [79]. It was demonstrated that the surface-bound affimer proteins are highly specific, differentiating between their target IgG and other closely related IgG subclasses [80]. Another class of synthetic, non-antibody protein scaffolds, called “repebody,” was created based on the adaptive immune system of jawless vertebrates, such as lampreys and hagfish. These scaffold proteins are derived from the variable lymphocyte receptor (VLR) molecules, instead of immunoglobulin antibodies. VLRs (and afterwards the repebodies) consist of highly diverse leucine-rich repeat (LRR) modules. Each VLR is characterized by an assembly of repeating 20–29 residue LRR modules, each of which has a β-strand-turn α-helix structure, in a horseshoe-shaped solenoid fold [81,82]. Combining varying numbers of LRR modules in different orders, in a “LEGO style”, allows for creating controllable shapes and modulating the binding affinity for a target, providing a wide range of applications [83].

When discussing scaffold protein structures based on repeated units, it is worth mentioning DARPins (Designed Ankyrin Repeat Proteins), which are synthetic proteins based on repeating structural motifs (ankyrins). These motifs are found in natural proteins and are involved in protein–protein interactions. Ankyrin repeats typically consist of 33 amino acid residues and form a structural unit composed of a β-turn followed by two antiparallel α-helices. These units connect to each other to form a flexible, curved surface. Fc-specific DARPins can be generated by selecting from combinatorial libraries using ribosome or phage display methods. Although DARPins have high potential to be used in the affinity chromatography of IgGs, they have not become widely used as a routine tool for immunoglobulin purification, being more commonly used as a platform for advanced therapeutic and diagnostic applications, such as the development of new protein drugs and diagnostic tools [97].

Monobodies are synthetic binding proteins that are built using the tenth fibronectin type III (FN3) domain of human fibronectin as a molecular scaffold. This domain was described in detail in 1998 and was chosen as an alternative scaffold due to its structural similarity to antibody variable domains [86,87,88]. FN3 is a small monomer (94 amino acids) that is based on a beta-sandwich fold composed of seven strands connected by six loops. One more, quite exotic, group of binding scaffolding proteins, nanobodies, are the smallest known natural antigen-binding fragments. They represent a single recombinant variable domain of the heavy chain (VHH) obtained from the heavy chain antibodies of animals of the *Camelidae* family (camels, llamas, and alpacas). Currently, the main focus is on increasing the capacity of nanobody-based platforms. It has been demonstrated that the creation of tandem (dimeric or trimeric) nanobody structures can significantly enhance the binding capacity of resins when compared to the monomeric form [98].

The short peptide ligands, which are often much cheaper and easier to produce, are of great interest now. Among them, dendrimeric peptides like PAM (Protein A Mimetic) and its protease-resistant D-PAM variant deserve special attention. These peptides are seen as a promising alternative to SpA and have been successfully used in commercial products [99]. Linear hexapeptides such as HWRGWV and its most popular counterparts HYFKFD and HFRRHL bind to the Fc fragment of antibodies and allow elution under mild acidic conditions (pH 4) [89,90]. Cyclic peptides, due to their ring-like structure, achieve increased stability and a much higher affinity than their linear precursors [91]. Another group of potential ligands for immunoglobulin chromatography is synthetic low-molecular-weight ligands that exclude short peptides. This group includes a wide variety of structurally simple organic molecules that were discovered through screening chemical libraries. These compounds often mimic well-known bacterial proteins, such as SpA and SpG, and are based on structures such as the triazine ring scaffolds [94] or the Ugi scaffold [95]. Aptamers, short single-stranded nucleic acids, are another class of innovative molecules that can fold into unique three-dimensional shapes and bind to target proteins with high affinity and specificity [96]. Finally, mixed-mode ligands (MML), or multimodal ligands, are one of the most powerful and advanced areas of recent research. These are chemical compounds designed specifically for use in chromatographic systems, which combine several different types of interactions simultaneously. These ligands have unique properties that allow them to bind to target molecules, such as proteins and other biological polymers, through multiple mechanisms, including hydrophobic, hydrophilic, and ion-exchange interactions. This provides increased selectivity and efficiency in separation processes, competing even with classical affinity chromatography of SpA [92,93].

Some other types of affinity ligands are being developed that bind to different subdomains of the Fab region, in contrast to those that target the Fc fragment of an antibody. These ligands can be useful for capturing antibody fragments that lack the Fc region. For instance, CaptureSelect CH1-XL specifically binds to the CH1 domain of the heavy chain, while KappaSelect and LambdaFabSelect bind to the constant region of kappa and lambda light chains in human IgGs, respectively. Some examples of these ligands are mentioned in Table 5.

Overall, there is a clear trend towards the design of smaller, more stable, and cost-effective synthetic ligands for specific applications. These molecules can be used in high-throughput bioprocessing and medical therapy, among other areas.

## 8. Conclusions and Future Perspectives

The market for affinity sorbents, which are used in the purification of immunoglobulins, is currently experiencing steady growth, driven by the expansion of the biotechnology industry. In large-scale antibody production in biotechnology, the purification process consumes a significant amount of time and resources. Despite the use of multiple steps, affinity chromatography remains the cornerstone of the purification process due to its high selectivity and relative simplicity.

Today, industrial antibody purification is still mainly based on bacterial IBPs. With that, most technologies are still based on the protein A recombinant variants. According to various projections, the market size for affinity resins based on protein A is expected to increase by at least twofold in the next five years. At the same time, commercial manufacturers offer a wide range of affinity resins with the required characteristics of species specificity and stability. Some commercially available resins are listed in Table 5.

The most significant areas of development for novel affinity sorbents continue to include the creation of versatile sorbents for purifying various types of antibodies, with improved binding properties and stability during regeneration. The last involves operating over a wide range of pH levels, being alkaline-stable, and preventing ligand loss from the surface of the support material. Other requirements include extending the lifespan of the sorbents and integrating them into a continuous purification process. In perspective, advances in combinatorial protein engineering and screening methods should lead to the development of novel classes of affinity ligands with desired properties. In the future, innovative rational design techniques and library screening approaches may open up new avenues for creating ligands with enhanced affinity, specificity, stability, and reduced manufacturing costs.

## Figures and Tables

**Table 1:** Properties of natural bacterial protein-based ligands.

Ligand	Origin	Ig Classes	IgG Species Specificity	Target	The Number of IgG-Binding Domains in wt Strains	Features	Ref.
Protein A (SpA)	*Staphylococcus aureus*	IgGs; human IgM, IgE, IgA (low affinity)	Human (except IgG3 subclass), mouse (except IgG1), rat (IgG2c), cow (IgG2), goat (IgG2), sheep (IgG2), rabbit.	Fc-part (CH2–CH3 regions), Fab-part (VH3, lower affinity)	5	The most extensively researched; the most commercially successful. The modified domain B is the most popular choice for market promotion.	[7,22,23]
Protein G (SpG)	*Streptococcus* spp.	IgG, IgY (chicken); does not bind to human IgM, IgD, IgA	A wide range of IgGs of all subclasses: human, mouse, rat (except IgG2b), cow, goat, sheep, and rabbit.	Fc-part (CH2–CH3 regions), Fab-part (CH1, lower affinity)	2–3	N-terminal domains of the wild-type protein G bind human serum albumin	[22,24,25,26]
Protein L (PpL)	*Peptostreptococcus magnus*	IgG, IgM, IgA, IgE, IgD	Human (all Ig classes); mouse, rat, pig IgGs, etc.	Fab-part (variable domains (VL) of kappa-type light chains); does not bind to lambda light chains and Fc-part	4–5	Antibody fragments that lack an Fc region (Fab, scFv, dAb) can be captured	[27,28,29]
Protein M	*Mycoplasma genitalium*	IgG, IgA, IgM	Human (IgG); murine, rat, rabbit, goat, and bovine IgGs.	Conserved regions of variable domain (Fv) of the kappa and lambda light chains	NS ^2^	Antibody fragments that lack an Fc region (Fab, F(ab′)_2_, scFv) can be captured; dissociation of the complex between IgG and the wild-type protein M is extremely slow	[30,31]
Hybrid Proteins ^1^	AG, LG, LA, AGL	-	Maximum specific IgG binding.	Variable	Variable	Versatility and flexibility	[32,33,34,35]

^1^ Recombinant protein ligands based on combinations of various affinity bacterial proteins; ^2^ NS—not specified.

**Table 2:** The main improvements in protein A-based affinity ligands.

Ligand	Modifications	Improvements	Ref.
Mutations			
Z domain (derived from B domain)	Gly29Ala point mutation in domain B	Alkaline stability (removes Asn–Gly cleavage site); eliminates Fab (VH3) binding; enables elution at higher pH	[18,41]
Z domain	Asn23Thr, Phe30Ala, Asn23Thr/Phe30Ala point mutations	Increased alkaline stability	[45]
Z domain	Combined point mutations Asn11Thr/Ala12Trp/Asn23Thr/Gln32Thr/Lys35Arg	Improved thermodynamic stability	[23]
Z domain	Gln10His/Asn11His double mutation	High pH sensitivity, thermal stability and alkaline tolerance, milder elution conditions (up to pH 5.6)	[46]
Z domain (Z4)	His18Ser, His18Ser/Asn28Ala point mutations	Milder elution pH (on average >0.5 pH units)	[27,43]
Z domain (Z_Ca_)	Insertion of Ca^2+^-binding loop between helices 2 and 3 (Z domain)	Calcium-dependent binding; elution with EDTA at neutral pH	[47]
Z domain	6-glycine or 4-glycine insertion between helix 2 and helix 3 of Z domain	Domain destabilization; enables milder elution conditions	[48]
C domain	Gly29Trp or Gly29Ala point mutations	Increased alkaline stability	[49]
C domain	Gly29Ala/Ser33Glu, Gly29Ala/Asp36Arg point mutations	Eliminates VH3-mediated Fab binding; retains alkaline resistance	[50]
Other improvements			
C domain	G46C Point mutation to cysteine	Allows oriented immobilization; improves alkaline tolerance	[51]
SpA (Thermal Responsive Protein A, TRPA)	Point mutations in hydrophobic core of the protein	Binding at 2–10 °C and elution at 40 °C at the neutral pH	[52]
Domain multimerization (tetramer to octamer of B, C or Z)	Genetic fusion of 2–8 identical domains	Potential increase in binding capacity	[13,15,38,40,42,43,44]
SpA (dextran-grafted proteinA)	Coupling protein A to a dextran layer on agarose	3D ligand surface distribution, improving antibody accessibility	[53]
SpA or Z domain	Oriented immobilization on the surface via carboxyl terminus of Z domain, via site-specific covalent conjugation.	Minimization of steric hindrance during interaction with mAb	[38,54,55,56]
Linker engineering	Insertion of flexible or rigid linker sequences between domains	Potentially improves domain accessibility and binding capacity	[15,57,58]

**Table 3:** Engineered versions of protein G-based affinity ligands.

Ligand	Modifications	Improvements	Ref.
Recombinant SpG from *Streptococcus* strain G148 expressed in *Escherichia coli*	The regions on the gene corresponding to the albumin-binding domains and the Fab-binding region were deleted by site-directed mutagenesis.	Protein G molecule binds only the Fc portion of IgG, but neither the Fab region nor albumin	[61]
Recombinant SpG from *Streptococcus* strain G148 expressed in *Escherichia coli*	Amino acid known to be susceptible towards high pH (asparagine) was substituted for less-alkali-susceptible residues (alanine) in the positions 7 and 36 of the C2 immunoglobulin-binding domain	Increased alkaline stability at the retained secondary structure and high affinity for the Fc fragment of IgG	[62]
SpG mutant	Ala24Glu, Lys28Arg, and Val29His point mutations	5-fold increase in affinity for human IgG	[63]
SpG mutant	Ala24Glu, Lys28His, Val29His, Asn35Glu point mutations	Switch in binding affinity by 50-fold when switching from pH 5.6 to pH 8.2	[64]
Protein-G-A1	8-point mutations at positions 18, 20, 24, 40, 41, 42 and 43	100-fold increase in affinity for the Fab scaffold compared to the wt-type domain	[65]
ProtG^N478R^	Asn478Arg mutation	Loss of Fab binding, no antibody cross-linking	[25]

**Table 4:** Bacterial IBPs-based recombinant hybrid ligands.

Ligand Name	Structure	Binding	Ref.
AG	5 domains (E, D, A, B, C) of SpA + 3 domains (C1, C2, C3) of SpG; 2 Z-domains (based on B-region) of SpA + 3 domains (C1, C2, C3) of SpG	All human IgG subclasses including IgG3 via Fc domain	[32]
AG	Full-length SpA and SpG proteins connected with n(DDAKK) linker (*n* = 0–6)	All human IgG subclasses via Fc and Fab regions	[35]
LG	4 B-repeats (B1, B2, B3, B4) of PpL + 2 C-repeats (C1, C2) of SpG	Large majority of intact human Igs via Fc region and Ig κ-light chains	[72,73]
LA	4 PpL domains + 4 SpA domains	All human IgG subclasses via Fc region and κ light-chain-binding domains	[34]
AGL	Full-length SpA (5 domains) + SpG (2 domains) + PpL (5 domains)	Maximum specific IgG binding (binds to human, mouse, rat cow, goat, sheep, rabbit, guinea pig, pig, dog and cat IgG)	[74]
AGL	Modified B domain of SpA + modified C2-domain of SpG + modified B3-domain of PpL	NS ^1^	[75]

^1^ NS—not specified.

**Table 5:** Contemporary commercial affinity resins available on the global market.

Resin	Manufacturer	Ligand	Ig Classes/Subclasses	Matrix	DBC_10_ (mg/mL)/RT (min)	Elution, pH	CIP ^1^ Treatment Alkaline Stability (NaOH)
Protein A-based resins							
rProtein A Sepharose FF	Cytiva (Marlborough, MA, United States)	Rec. ^2^ SpA	“+” ^3^ Human IgG1, IgG2, IgG4; mouse IgG2a, IgG2b, IgG3 “−” ^3^ Human IgG3; mouse IgG1	4% HCL ^4^-agarose	~35 mg human IgG/mL resin (3 min)	4.0	NR ^5^
Mabselect SuRe	Cytiva (Marlborough, MA, United States)	Rec. engineered alkali-tolerant SpA-derived ligand (Z-type tetramer)	NS ^6^	HCL-agarose	~35 mg hIgG/mL resin (2.4 min)	3.0	0.1–0.5 M
MabSelect SuRe LX	Cytiva (Marlborough, MA, United States)	Rec. engineered alkali-tolerant SpA-derived ligand (Z-type tetramer)	NS	Rigid, HCL-agarose	~60 mg human IgG/mL resin (6 min)	3.0–3.6	0.1–0.5 M
MabSelect PrismA™	Cytiva (Marlborough, MA, United States)	Rec. engineered alkali-tolerant SpA-derived ligand (Z-type hexamer)	NS	HCL-agarose	~65 mg polyclonal IgG/mL resin (4 min); ~80 mg polyclonal IgG/mL resin (6 min)	3.0–3.6	0.5–1.0 M
RUselect-A	GreenVan (Moscow, Russia)	Rec. engineered SpA (B-type dimer)	“+” Monoclonal and polyclonal antibody	6% HCL-agarose	50–55 mg/mL (10 min)	<4.0	0.1 M
RUselect-P	GreenVan (Moscow, Russia)	Rec. engineered SpA (B-type dimer)	“+” Monoclonal and polyclonal antibody	Polymethacrylate	45–50 mg/mL (10 min)	<4.0	0.1–0.5 M
Monofinity A	GenScript Biotech (GenScript Biotech, Piscataway, NJ, United States; Nanjing, Jiangsu, China)	Rec. alkali-tolerant SpA	“+” IgG from several species of mammals	4% HCL-agarose	>30 mg IgG/mL resin	3.0	0.1–0.5 M
Unosphere SuPra	Bio-Rad (Hercules, CA, United States)	Rec. SpA	“+” Monoclonal and polyclonal antibody	HCL-polymer	30 ± 3 mg/mL at 150 cm/hr; 25 ± 2 mg/mL at 300 cm/hr	3.0	0.1 M
ProSep^®^ Ultra Plus	Merck (Burlington, MA, United States)	Rec. native SpA	NS	Porous glass	>50 mg/mL (3–6 min), dynamic; ≥67 mg/mL (hIgG), static	NS	NR
Eshmuno^®^ A	Merck (Burlington, MA, United States)	Rec. SpA, derived from C domain of native SpA (C-type pentamer)	NS	Hydrophilic polyvinylether	DBC_5_: 40–55 mg/mL (3–6 min)	NS	0.1 M
POROS™ MabCapture™ A (MabCapture™ A Select)	Thermo Fisher Scientific (Waltham, MA, United States)	Rec. SpA	NS	CL ^7^-poly(styrene-divinylbenzene)	DBC_5_: ≥ 37 mg/mL	2.0–3.0	NR
MabCaptureC™ High Capacity Protein A Resin	Thermo Fisher Scientific (Waltham, MA, United States)	Rec., modified SpA (C domain based)	NS	CL-agarose	58 mg/mL 5 min); 80 mg/mL (10 min)	2.0–3.0	0.2 M
Toyopearl AF-rProtein A HC-650F	Tosoh Bioscience (Tokyo, Japan)	Rec., modified SpA (Y-type hexamer)	NS	Toyopearl HW-65F polymer base bead	>65 mg/mL (5 min); >50 mg/mL (2 min)	3.0	0.1 M
UniMab 50HC/UniMab EXE	NanoMicro Technology (Suzhou, Jiangsu, China)	Alkaline-stabilized rec. SpA	Monoclonal and polyclonal antibodies, bispecific antibodies, and Fc fusion constructs	Polymethacrylate	~50–65 mg/mL of human IgG (6 min)	NS	0.1–0.5 M
NMab Pro	NanoMicro Technology (Suzhou, Jiangsu, China)	Engineered SpA	NS	HCL-agarose	65–80 mg/mL (5 min)	NS	0.1–0.5 M
Protein G-based resins							
Protein G Resin	GenScript Biotech (Piscataway, NJ, United States; Nanjing, Jiangsu, China)	Rec. streptococcal protein G lacking the albumin-binding sites	“+” Most mammalian IgGs, including human IgG3, mouse IgG1, rat IgG2a; “−” Human IgM, IgD, IgA	4% HCL-agarose	>20 mg human IgG/mL resin	2.5	NS
NMab ProtG	NanoMicro Technology (Suzhou, Jiangsu, China)	Rec. protein G	“+” Human IgG1, IgG2, IgG3, IgG4, Fab; Mouse IgG1, IgG2a, IgG2b, IgG3; Rat IgG1, IgG2a, IgG2b, IgG2c; Rabbit, donkey, chicken IgGs; sheep (IgG1, IgG2), cow(IgG1, IgG2)	HCL-agarose	55–65 mg/mL human IgG (5 min)	3.0	NR
Pierce™ Protein G (Pierce™ Protein G Plus)	Thermo Scientific™/Pierce (Waltham, MA, United States)	Rec. protein G	“+” Most mammalian IgGs, especially for certain subclasses including human IgG3, mouse IgG1 and rat IgG2a; “−” Human IgM, IgD and IgA	6% CL beaded agarose	11–15 mg human IgG/mL (>20 mg human IgG/mL)	2–3	NR
Protein L-based resins							
Capto L	Cytiva (Marlborough, MA, United States)	Rec. protein L	“+” Fabs, single-chain variable fragments (scFv), domain antibodies (dAbs)	Rigid, HCL-agarose	~25 mg/mL (Fab, 4 min)	2.0–3.5	15 mM
MabSelect™ VL	Cytiva (Marlborough, MA, United States)	Alkaline-stabilized protein L-derived ligand	“+” Kappa light chain, Fab, ScFv, Dab; human lgG1, lgG2, lgG3, lgG4, lgA, lgD, lgE, lgM; mouse lgG1, lgG2a, lgG2b, lgG3, lgM; rat lgG1, lgG2a, lgG2b, lgG2c; pig IgG; dog IgG (weak); “−” Lambda light chain, heavy chain; cow (IgG1, IgG2), goat (IgG1, IgG2), sheep (IgG1, IgG2), chicken IgG	HCL-agarose	~70 mg human IgG/mL (6 min); ~60 mg human IgG/mL (4 min)	3.1–3.5	0.1 M
Protein L Resin	GenScript Biotech (Piscataway, NJ, United States; Nanjing, Jiangsu, China)	Highly purified protein L	“+” κ light chain; Fab, Scfv, Dab; human lgA, lgD, lgE, lgG1, lgG2, lgG3, lgG4, lgM; mouse lgG1, lgG2a, lgG2b, lgG3, lgM; rat lgG1, lgG2a, lgG2b, lgG2c; dog, hamster, horse, koala, monkey, pig, rabbitIgGs; guinea pig lgG1, lgG2. “−” λ light chain, heavy chain; cow, goat, sheep IgGs; avian egg yolk lgY; chicken IgY	4% agarose	Static binding capacity ≥ 15 mg human IgG/mL resin	2.5	NS
Pierce™ Protein L	Thermo Scientific™/Pierce (Waltham, MA, United States)	Rec. protein L	“+” All classes of Ig (IgG, IgM, IgA, IgE and IgD), single-chain variable fragments (Scfv) and Fab fragments	6% CL beaded agarose	~5–10 mg human IgG/mL	2–3	NR
NMab ProtL	NanoMicro Technology (Suzhou, Jiangsu, China)	Rec. protein L	“+” Kappa light chain, Fab, ScFv, Dab; human lgG1, lgG2, lgG3, lgG4, lgA, lgD, lgE, lgM; mouse lgG1, lgG2a, lgG2b, lgG3, lgM; rat lgG1, lgG2a, lgG2b, lgG2c; pig IgG; dog IgG (weak); “−” Lambda light chain, heavy chain; cow (IgG1, IgG2), goat (IgG1, IgG2), sheep (IgG1, IgG2), chicken IgG	HCL-agarose	~75 mg/mL (Fab, 5 min)	2.5	NR
Hybrid protein-based resins							
Pierce™ Protein A/G Agarose	Thermo Scientific™/Pierce (Waltham, MA, United States)	Rec. AG (4 SpA domains + 2 SpG domains)	“+” All human IgG subclasses; IgA, IgE, IgM (somewhat); IgD (to a lesser extent)	6% CL beaded agarose (CL-6B)	Binding capacity: 9 mg mouse IgG or >7 mg human IgG/mL	2–3	NR
Pierce™ Protein A/G Plus Agarose	Thermo Scientific™/Pierce (Waltham, MA, United States)	Rec. AG (4 SpA domains + 2 SpG domains)	“+” All human IgG subclasses; IgA, IgE, IgM (somewhat); IgD (to a lesser extent)	6% CL beaded agarose (CL-6B)	Binding capacity: 27 mg mouse IgG or >50 mg human IgG/mL	2–3	NR
Pierce™ Protein A/G UltraLink™ Resin	Thermo Scientific™/Pierce (Waltham, MA, United States)	Rec. AG (4 SpA domains + 2 SpG domains)	“+” IgG from several mammalian species, especially most subtypes of human and rabbit IgG	Polyacrylamide (UltraLink Resin)	Binding capacity: >20 mg human IgG/mL	2.5–3.0	NR
Immobilized Protein A/G	G-Biosciences (MO, United States)	Rec. AG (4 SpA domains + 2 SpG domains), lacks the albumin-binding sites.	“+” Most mammalian IgGs excluding chicken IgG; “−” IgA, IgM or serum albumin	6% HCL-agarose	Binding capacity: 38 mg human IgG/mL resin; >20 mg sheep IgG/mL resin	3.0	NS
Fab fragment sites binding ligands based resins							
CaptureSelect™ CH1-XL	Thermo Fisher Scientific (Waltham, MA, United States)	Rec. single-domain llama heavy chain-based antibody fragments (VHH) created in the yeast *Saccharomyces cerevisiae*	“+” CH1 domain; human Fab- and Fab2 fragments and human IgG (all four subclasses)	Epoxide-activated agarose	19 g of polyclonal human Fab/L of matrix (2 min)	4.0–4.5	10 mM
CaptureSelect™ KappaXP	Thermo Fisher Scientific (Waltham, MA, United States)	Rec. single-domain Camelidae heavy chain-based antibody fragments (VHH) created in the yeast *Saccharomyces cerevisiae*	“+” Human Fab- and Fab2 fragments and Igs with a kappa light chain; “−” Bovine antibody	Epoxide-activated agarose	20 g of polyclonal human Fab/L of matrix and 35 g of polyclonal human IgG/L of matrix (6 min)	3.5	75–100 mM
CaptureSelect™ LambdaXP	Thermo Fisher Scientific (Waltham, MA, United States)	Rec. single-domain Camelidae heavy chain-based antibody fragments (VHH) created in the yeast *Saccharomyces cerevisiae*	“+” Human Fab- and Fab2 fragments and Igs with a lambda light chain “−” Fc-region or kappa light chains	Epoxide-activated agarose	20 g of polyclonal human Fab/L of matrix and 35 g of polyclonal human IgG/L of matrix (6 min)	3.0	50–80 mM
Purolite™ Praesto™ 70 CH1	Ecolab (Purolite) (King of Prussia, PA, United States)	Rec. protein, alkali-tolerant (NS)	“+” CH1 domain of the heavy chain of human IgG	HCL-agarose	40 g/L mAb (IgG1) Fab (6 min)	2.8	0.1 M
KappaSelect	Cytiva (Marlborough, MA, United States)	Rec. protein, produced in *Saccharomyces cerevisiae*	“+” Human Fab (kappa) fragments	HCL-agarose	Binding capacity: 15 mg Fab/mL resin	2.5–3.0	10 mM
LambdaFabSelect	Cytiva (Marlborough, MA, United States)	Rec. protein, produced in *Saccharomyces cerevisiae*	“+” Human lambda Fab fragments	HCL-agarose	~20 mg Fab/mL resin (4 min)	3.5	10 mM

^1^ CIP—Cleaning-In-Place treatment. ^2^ Rec.—recombinant. ^3^ “+” binds, “−”does not bind. ^4^ HCL—Highly cross-linked (agarose). ^5^ NR—not recommended. ^6^ NS—not specified. ^7^ CL—Cross-linked.

**Table 6:** Alternative affinity ligands for immunoglobulins.

Name	Origin	Size, kDa	Binding Site	Kd	Features	Ref.
*Alternative Scaffold Proteins*						
Affibody	SpA Z-domain-based molecules	~6	Fc-site		A synthetic bacterial-derived ligand based on the Z (B-derived) domain of SpA.	[76]
Affitins	Artificial affinity proteins designed from Sac7d (*Sulfolobus acidocaldarius*) or Sso7d (*Sulfolobus solfataricus*) archaeal proteins	~7	Fc-site	Nanomolar and subnanomolar ranges	Exceptional resistance to extreme conditions, including high temperatures (up to 90–100 °C), a wide pH range (0–12), and the presence of denaturing agents or high concentrations of salts and organic solvents.	[77]
Affimers	Artificial affinity proteins designed from human stefin A protein or plant phytocystatin consensus	~12–14	Variable regions	Picomolar to micromolar range	Cystatin fold with two variable loops to create specific binding sites; ultra-high thermal stability in a wide range of conditions	[78,79]
Nanobodies	Recombinant camelid antibody fragments, consisting of a single variable domain (VHH)	~15	Anti-Fc VHH	Subnanomolar range	Remarkable physicochemical stability, high solubility and improved tissue penetration ability, along with a compact structure (nanosizes) and low molecular weight. Low immunogenicity.	[84,85]
Monobodies	Human fibronectin (tenth fibronectin type III (FN3) domain)	~10	Antibody variable (VH) domains	Nanomolar range	Compact and easy to fit. Have clear potential for industrial applications.	[86,87]
Repebodies	Synthetic non-antibody protein scaffold derived from variable lymphocyte receptors (VLR) of jawless vertebrates composed of highly diverse leucine-rich repeat (LRR) modules.	~20	Fc-site	10^−7^ M for the highest affinity repebody ligands	Mild elution conditions (pH 4.0) and exceptional resistance to alkaline treatment (incubation with 0.5 M NaOH for 15 days without any loss of activity). Have clear potential for industrial applications.	[81,82]
*Short Peptide Ligands*						
PAM and D-PAM	18–86Synthetic peptides	12 amino acids	Specific regions of the antibody molecule, most often the conservative Fc fragment	Micromolar to nanomolar range	Can be obtained through cheaper chemical synthesis. Stable and flexible to fit different purposes. Can be scaled up for industrial use.	[18,88]
Hexapeptides (HWRGWV and others)		6 AA				[89,90]
Cyclic peptides		Variable				[91]
*Others*						
Mixed-Mode Ligands (MML)	-	-	-	-	Different types of interactions are combined to create a variety of combinations.	[92,93]
Synthetic Low-Molecular-Weight Ligands	Triazine-based scaffolds, Ugi scaffold, etc.	Up to 1000 Da	Variable	Variable	High chemical stability due to the non-biological nature.	[94,95]
Aptamers	Short single-stranded nucleic acids (DNA or RNA)	20–80 nucl. bases	Most often the conservative Fc fragment	Not specified	No immunogenicity.	[96]

## Data Availability

No new data were created or analyzed in this study. Data sharing is not applicable to this article.

## Abbreviations

The following abbreviations are used in this manuscript:

IBP	Immunoglobulin-binding proteins
SpA	Staphylococcal protein A
SpG	Streptococcal protein G
PpL	Peptostreptococcal protein L
IgG	Immunoglobulin G
IgA	Immunoglobulin A
IgM	Immunoglobulin M
mAbs	Monoclonal antibodies
RT	Residence time
DBC	Dynamic binding capacity
